# Making Friends: Social Attraction in Larval Green and Golden Bell Frogs, *Litoria aurea*

**DOI:** 10.1371/journal.pone.0056460

**Published:** 2013-02-12

**Authors:** Stephan T. Leu, Martin J. Whiting, Michael J. Mahony

**Affiliations:** 1 School of Environmental and Life Sciences, University of Newcastle, Newcastle, New South Wales, Australia; 2 Department of Biological Sciences, Macquarie University, Sydney, New South Wales, Australia; University of Western Ontario, Canada

## Abstract

Socio-ecological models combine environmental and social factors to explain the formation of animal groups. In anurans, tadpole aggregations have been reported in numerous species, but the factors driving this behaviour remain unclear. We conducted controlled choice experiments in the lab to determine whether green and golden bell frog (*Litoria aurea*) tadpoles are directly attracted to conspecifics (social factors) in the absence of environmental cues. Using repeated measures, we found that individual tadpoles strongly preferred associating with conspecifics compared to being alone. Furthermore, this preference was body size dependent, and associating tadpoles were significantly smaller than non-associating tadpoles. We suggest that small tadpoles are more vulnerable to predation and therefore more likely to form aggregations as an anti-predator behaviour. We demonstrate that tadpoles present an ideal model system for investigating how social and ecological factors influence group formation in vertebrates.

## Introduction

Group formation in animals has evolved many times independently in response to a wide range of selective pressures [1]. Understanding the ultimate factors and proximate mechanisms influencing group formation and ultimately sociality, is a major challenge in evolutionary biology. Both environmental and social factors have been identified to confer a fitness advantage and to influence the formation of animal aggregations and have been combined into socio-ecological models. Important environmental factors include the abundance, distribution, and quality of resources, such as discrete food patches [2], nest sites [3] and refuges [4].

The most commonly recognized social factors and benefits of being in a group include dilution of predation risk [5], increased vigilance and early predator detection [6], [7], thermoregulation [8] and enhanced feeding through the use of social information [9], [10]. Nevertheless, there are also costs to group formation which are often proportional to group size and include increased within-group competition [11], home range size and travel distances [12], and susceptibility to parasites and pathogens [13], [14]. These costs and benefits, in a species-specific combination, selected for the independent evolution of group living in numerous taxa [15].

Tadpole schooling has evolved multiple times in distantly related taxa and occurs in at least 10 families [16]. Much of our understanding of the causes and consequences of tadpole schooling is based on observations in the field and a number of laboratory studies [16]–[19]. A key finding suggesting the importance of social cues for group formation is that tadpoles of *Bufo americanus* and several other species are capable of kin recognition and preferentially school with siblings [19]–[22]. Interestingly, the majority of aggregating species breed in ephemeral ponds [16], indicating that tadpoles may aggregate at scarce food resources. Conversely, it has been shown that daylight induces group formation [23], and water clarity increases group size and decreases inter-individual distances [24], which both suggest a spacing pattern that enhances anti-predator behaviour. Social foraging [25] and the use of social information to find food [26] have also been suggested as important social factors selecting for the formation of tadpole aggregations.

Taken collectively, the relative importance of ecological and/or social factors for the formation of tadpole aggregations is as yet unclear. The green and golden bell frog (*Litoria aurea*) is a large endangered Australian frog (adult snout-vent length 55–90 mm) [27]. It is adapted to breeding in permanent ponds [28] and its tadpoles (snout-vent length 3–30 mm from hatching to metamorphosis) [27] can be under strong predation pressure, for example by the mosquitofish (*Gambusia holbrooki*) [29], [30]. *Litoria aurea* tadpoles have occasionally been found in aggregations in the wild [27]. We tested whether tadpoles form groups on the basis of social conspecific attraction in the absence of environmental resources.

## Materials and Methods

### Ethics statement

All methods and procedures were formally approved by the University of Newcastle Animal Care and Ethics Committee (approval no A-2011-150) in compliance with the Australian Code of Practice for the use of animals for scientific purposes.

### Tadpole husbandry

Adult green and golden bell frogs were collected from Kooragang Island in the Hunter Estuary in New South Wales (−32°51′48″/151°43′55″) during 2010 as part of an independent breeding project. For the experiment reported here, we randomly selected tadpoles from two clutches laid by two different females in captivity, in December 2011. Tadpoles were housed in two mixed sibship groups, with both group sizes varying repeatedly over time between 25–100 individuals (approximately 5–20 tadpoles/L water). Social experience through raising tadpoles in different group sizes does not influence their propensity to aggregate [31]. Tadpoles were maintained in aged carbon-filtered water at room temperature on the natural light cycle (light-dark cycle of 14∶10 h) and they were fed three times/week with sprinkles of Goldfish Flake Plus (Marine Master). Water was changed as required, on average every four days (range 1–7, *N* = 9). Tadpoles were returned to the breeding project after the experiment.

### Tests of social attraction

**Figure 1:**
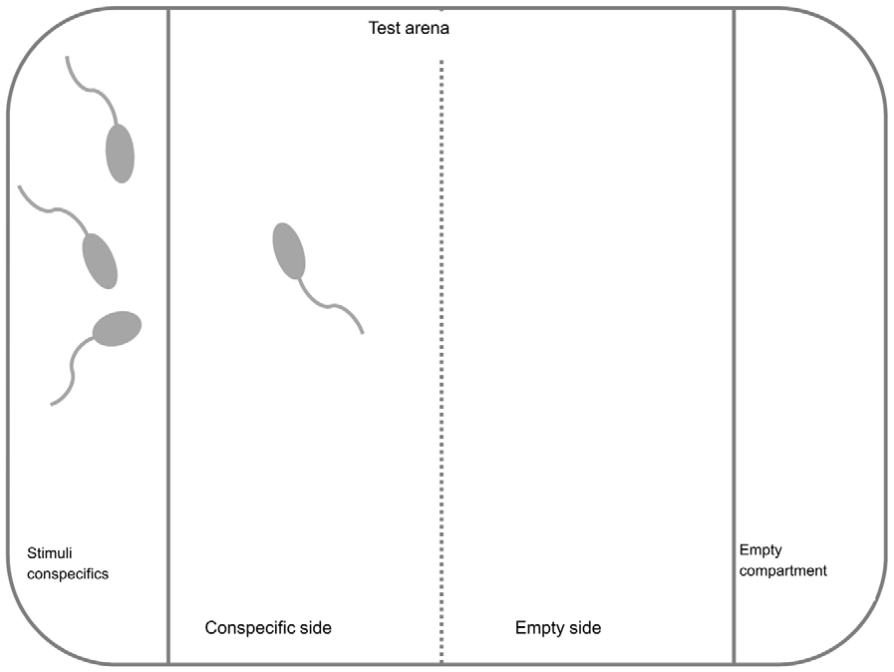
Test arena design. Arena with adjoining compartments on either side to hold conspecifics (test stimulus). A line on the base of the chamber (here dashed) divided the arena into halves.

We tested a total of 48 tadpoles. Each tadpole was tested once, and all were at Gosner developmental stage 25 [32], [33]. The study group was divided into two groups of 24 tadpoles, which were tested on consecutive days at similar times when tadpoles were active. Tadpoles were fed within 24 hours prior to the experiment to minimize the influence of the nutritional state on grouping behaviour. We used six test chambers at a time, which were visually isolated from each other. Each test chamber (figure 1) was partitioned into three compartments, the rectangular test arena (9.0×9.2×5.0 cm) in the middle and one adjoining compartment (2.8 cm×9.2×5.0 cm) on opposite sides. The test arena was divided in half again by a line on the base of the arena. We used three conspecifics as stimuli in each apparatus. Different stimuli tadpoles were used during the second experimental day. On each day focal and stimuli tadpoles were taken from one of the two mixed sibship groups. Focal and stimuli tadpoles were not size matched. Partitioning walls were transparent to allow visual contact between stimuli and focal tadpoles. Each day, we filled the six test chambers with water from a container that held all 42 tadpoles (24 focal tadpoles + 6×3 conspecific stimuli tadpoles) for 15 minutes, to minimize differences in tadpole chemical cues between trials. The partitioning walls were not watertight and tadpole-borne chemical cues could diffuse from the conspecific compartment into the arena. Visual and chemical cues were available to the focal tadpoles. We controlled for spatial preference in focal tadpoles by haphazardly assigning conspecifics to three left and three right compartments in the six test chambers used at a time. The water level in the test chambers was 2 cm. *L. aurea* tadpoles can occur in shallow water and often swim near the water surface [27]. Furthermore, following similar experiments [17], [20], [26], we chose this relatively low water level to allow us to clearly determine the position and distance of the focal individual relative to conspecifics. After introducing a focal tadpole into each arena we allowed them two minutes to acclimate, inspect the arena with its two adjoining compartments, and make a decision. We then took four photographs at two minute intervals from behind a blind to establish the tadpole's position. At the end of each experimental day we measured the body size (snout-vent length) of each tadpole to the nearest 0.1 mm.

### Data analyses

We used the photographs to score whether tadpoles were present on the half of the test arena adjacent to the compartment with conspecifics or the empty compartment. We discarded five trials because they could not be accurately scored, leaving a total of 43 trials. Tadpoles were active and usually moved during the two minute interval between photographs. We used Cochran's Q test (an extension to the McNemar test) to ensure that repeated testing did not affect the decision making process in a systematic way [34], as the precision of decision making may improve with repetition [35], [36]. We then allocated trials to one of two categories: (1) focal tadpole present on the conspecific side three or four times out of the four measures; and (2) focal tadpole present on the empty side two, three or four times. We used a chi-square test to determine whether option (1) occurred more frequently than expected by chance and hence whether tadpoles were attracted to conspecifics. Expected frequencies were calculated using the probability mass function for binominal distributions. Finally, we used a two-sample *t*-test to investigate whether social attraction strength was a function of body size. Data were reflect square root transformed due to negative skew to meet the assumption of normality. We used an alpha level of 0.05 to denote significant results. Statistical analyses were performed in SPSS 17.0.2.

## Results

**Figure 2:**
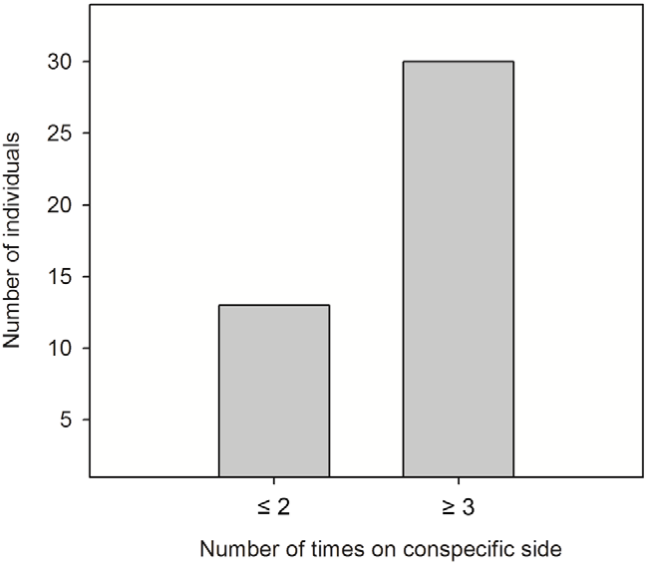
Social preference in *Litoria aurea* tadpoles. Number of individuals that repeatedly chose the side with conspecifics over the empty side.

**Figure 3:**
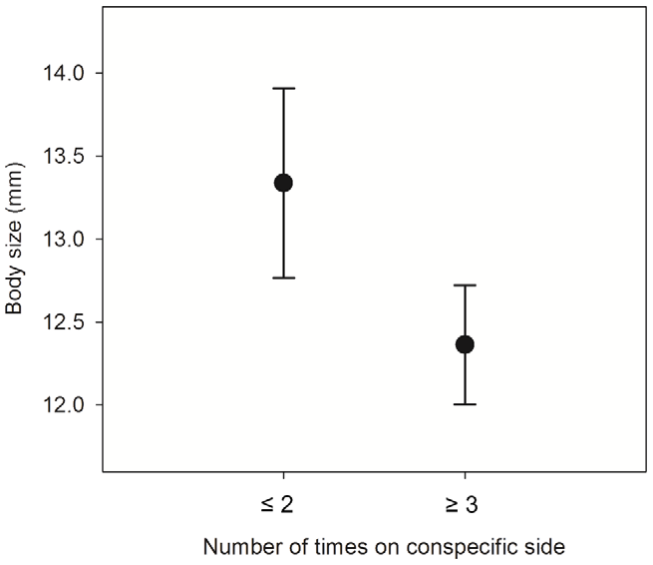
Mean (± SE) tadpole body size in relation to social preference. Body sizes (snout-vent lengths) are shown as untransformed data.

Whether tadpoles chose to be alone or to associate with conspecifics was not significantly different among trials (Cochran's Q test: Q_3_ = 6.66, *P* = 0.08), and neither increased nor decreased through the course of multiple trials. This indicated a high repeatability among trials. Significantly more tadpoles than expected by chance chose the compartment containing conspecifics at least three out of four times (category 1;Chi-square test: χ^2^_1_ = 29.69, *P*<0.0001; figure 2). Furthermore, social attraction was a function of body size. Tadpoles that were attracted to their conspecifics (category 1) were significantly smaller than tadpoles that showed no social attraction (category 2, unpaired *t*-test: *t*_41_ = −2.84, *P = *0.007; figure 3).

## Discussion

We conducted a relatively simple choice experiment in which isolated tadpoles had the option of spatially associating with an empty compartment or a compartment containing a group of three conspecific tadpoles. We found a very strong preference for the compartment containing conspecifics, which suggests that *Litoria aurea* tadpoles prefer to aggregate with conspecifics through social attraction. Whether visual or olfactory cues were used to locate the conspecifics remains to be determined. Alternatively, tadpoles may have avoided the empty compartment. However this is unlikely, because *L. aurea* tadpoles occupy structured habitats in the wild, yet have been observed to school [27].

Both environmental and social factors have been identified to select for the formation of animal aggregations. By conducting controlled experiments in the lab, we were able to exclude environmental factors such as food, temperature and light gradients that might influence tadpole grouping behaviour [37], [38]. This indicates that aggregation behaviour in *L. aurea* may be driven by social factors. One such social factor is the benefit of schooling in the presence of a predator [39] and/or when tadpoles detect chemical cues from a predator [40]. Predators may include larger conspecifics [41] and therefore represent another form of natural selection. This may result in relatively higher predation pressures on smaller individuals. Small tadpoles are most vulnerable to predation [42], and tadpoles generally become less vulnerable as they grow [43], because larger tadpoles are able to escape from predators more readily [44] or are too large for canibalistic conspecifics. However, during metamorphosis tadpoles are once again vulnerable because their emerging limbs may compromise locomotor ability [44]. Interestingly, we found a relationship between social attraction and tadpole size in *L. aurea* tadpoles. Individuals that were attracted to conspecifics were significantly smaller than tadpoles that showed no social attraction. This may indicate that schooling in *L. aurea* could be an anti-predator behaviour. This hypothesis requires empirical testing, especially since the tadpoles in our study were born in captivity and not exposed to predatory cues.

Another social route to tadpole schooling is the benefit of social foraging and the transfer of social information to obtain food. For example, tadpoles of the spadefoot toad *Spea multiplicata* have been shown to form a vortex in response to introduced food particles in feeding experiments [25]. It has been suggested that this collective behaviour allows access to food resources through agitating the pond substrate that otherwise would be inaccessible [25]. Similarly, *Bufo americanus* tadpoles use social information through the presence of conspecifics, to locate food patches, rather than chemical cues from the food source itself [26].

A third social route to schooling appears to be relatedness. The tadpoles of several species preferentially school with kin and remain in schools even in the absence of an immediate predatory threat [19], [20]. However, the mechanisms driving kin recognition and preference for siblings appear to be varied [16] and there is even debate that kin recognition might simply be a by-product of species and/or group member recognition and therefore not true kin recognition [45]. Nevertheless, a popular argument in support of kin-based schooling is that if distasteful tadpoles are preyed upon, this cost is balanced against the improved survival of siblings [20]. Hence, relatedness could be an alternative explanation for the observed association behaviour in *L. aurea*, which would indicate a genetic determination. However, we did not distinguish tadpoles on the basis of their clutch, so the effects of relatedness on schooling behaviour are difficult to gauge in our study.

There has been considerable debate and speculation about the factors and cues that drive group formation in tadpoles [16]. While environmental drivers appear to be important for some species, social reasons are equally important for other species. Our study establishes the existence of tadpole schooling in *L. aurea* and indicates the importance of social factors, but more research is required to distinguish between the possible functions of this behaviour. For example, whether *L. aurea* tadpoles are attracted to conspecifics as an anti-predator behaviour or because conspecifics signal the presence of food remains to be determined. Nevertheless, the observed inverse relationship between tadpole size and aggregation preference supports the notion of an adaptive anti-predator behaviour.

Given the significance of the tadpole phase for successful reproduction in frogs, we need more studies examining social and environmental factors that potentially drive schooling behaviour, in order to construct a general theory for the evolution of social aggregations in tadpoles. Tadpoles represent an ideal model system for the understanding of socio-ecological factors that drive group formation during a vulnerable life stage.
